# Impact of COVID-19 on antenatal care provision

**DOI:** 10.18332/ejm/121096

**Published:** 2020-05-07

**Authors:** Sarah Esegbona-Adeigbe

**Affiliations:** 1 Adult Nursing and Midwifery Studies, School of Health and Social Care, London South Bank University, London, United Kingdom

**Keywords:** COVID-19, antenatal care, maternal mortality, high-risk women

**Dear Editor,**

Antenatal care services have been affected by the COVID-19 pandemic despite the fact that pregnant women were placed in the ‘vulnerable group’ by the UK Government on 16 March 2020^1^. The risk of pregnant women contracting COVID-19 means that careful surveillance of their health is needed. Prevention and control of COVID-19 infection among pregnant women and the potential risk of vertical transmission have become a major concern^2^. However, the COVID-19 pandemic has led to maternity services adjusting how they provide antenatal care to pregnant women due to the government restrictions regarding social distancing, which has impacted on pregnant women’s access to routine antenatal care. The advice to all pregnant women from the Royal College of Obstetricians and Gynaecologists is to decide if the need for an antenatal appointment is greater than the risk of being exposed to COVID-19^1^. This means that women have to make the difficult decision of what antenatal care provision they require.

Pregnant women face additional challenges during social distancing because of their contribution to the workforce, as care givers, and the need to attend antenatal care^3^. Remote antenatal appointments are being offered by maternity units if a face-to-face consultation is deemed unnecessary after a telephone consultation. The concern is that women are being asked to negotiate services when the socioeconomic impact of COVID-19 may have detrimental effects on their psychological and physical wellbeing. Women’s rights to have optimum antenatal care are threatened; if they have an appointment that does not involve an ultrasound or laboratory test then they are simply told not to come to the hospital or visit a health centre^4^.

According to UK maternal mortality reports, women at particular risk of dying during pregnancy are Black, Asian, ethnic minority women, immigrants, victims of domestic violence and women of lower socioeconomic status^5^. Consideration of how high-risk women negotiate maternity services during the pandemic needs a careful review of their needs on an individual basis. This is particularly relevant for hard to reach women who, under usual circumstances, may not access or engage with maternity services. Women who inadequately utilise antenatal services are twice as likely to be at risk for maternal morbidity^6^. Disruption of maternity services and diversion of resources away from essential pregnancy care, because of prioritising the COVID-19 response, are expected to increase risks of maternal morbidity and mortality^7,8^. The long-term effects of maternal morbidity and mortality on families, societies and communities should not be underestimated. Consideration should be given to the need to provide appropriate antenatal care for highrisk women in the current pandemic. This can be achieved by proficient screening by maternity units to ensure women that need and should have face-to-face consultations are provided this service.
